# Diffusion of subsidized ACTs in accredited drug shops in Tanzania: determinants of stocking and characteristics of early and late adopters

**DOI:** 10.1186/1472-6963-13-526

**Published:** 2013-12-18

**Authors:** Peter S Larson, Prashant Yadav, Sarah Alphs, Jean Arkedis, Julius Massaga, Oliver Sabot, Jessica L Cohen

**Affiliations:** 1 Department of Epidemiology, School of Public Health, University of Michigan, 1415 Washington Heights, Ann Arbor, MI 48109-2029, USA; 2 The William Davidson Institute, University of Michigan, 724 E University Avenue, Ann Arbor, MI 48109, USA; 3 Ross School of Business, University of Michigan, 701 Tappan Avenue, Ann Arbor, MI 48109, USA; 4 School of Public Health, University of Michigan, 1415 Washington Heights, Ann Arbor, MI 48109, USA; 5 Results for Development Institute, 1100 15th Street, NW, Suite 400, Washington, DC, USA; 6 National Institute for Medical Research, P.O. Box 9653, Dar es Salaam, Tanzania; 7 Clinton Health Access Initiative, 383 Dorchester Avenue, Suite 400, Boston, MA 02127, USA; 8 Harvard School of Public Health, 677 Huntington Avenue, Boston, MA 02115, USA

**Keywords:** Malaria, ACT, Drug shops, Tanzania, Marketing, Product diffusion

## Abstract

**Background:**

Many households in sub-Saharan Africa utilize the private sector as a primary source of treatment for malaria episodes. Expanding access to effective treatment in private drug shops may help reduce incidence of severe disease and mortality. This research leveraged a longitudinal survey of stocking of subsidized artemisinin combination therapies (ACTs), an effective anti-malarial, in Accredited Drug Dispensing Outlets (ADDOs) in two regions of Tanzania. This provided a unique opportunity to explore shop and market level determinants of product diffusion in a developing country retail market.

**Methods:**

356 ADDOs in the Rukwa and Mtwara regions of Tanzania were surveyed at seven points between Feb 2011 and May 2012. Shop level audits were used to measure the availability of subsidized ACTs at each shop. Data on market and shop level factors were collected during the survey and also extracted from GIS layers. Regression and network based methodologies were used. Shops classified as early and late adopters, following Rogers’ model of product diffusion, were compared. The Bass model of product diffusion was applied to determine whether shops stocked ACTs out of a need to imitate market competitors or a desire to satisfy customer needs.

**Results:**

Following the introduction of a subsidy for ACTs, stocking increased from 12% to nearly 80% over the seven survey rounds. Stocking was influenced by higher numbers of proximal shops and clinics, larger customer traffic and the presence of a licensed pharmacist. Early adopters were characterized by a larger percentage of customers seeking care for malaria, a larger catchment and sourcing from specific wholesalers/suppliers. The Bass model of product diffusion indicated that shops were adopting products in response to competitor behavior, rather than customer demand.

**Conclusions:**

Decisions to stock new pharmaceutical products in Tanzanian ADDOs are influenced by a combination of factors related to both market competition and customer demand, but are particularly influenced by the behavior of competing shops. Efforts to expand access to new pharmaceutical products in developing country markets could benefit from initial targeting of high profile shops in competitive markets and wholesale suppliers to encourage faster product diffusion across all drug retailers.

## Background

Despite the successful development of effective medications to treat health conditions such as malaria, diarrheal disease, HIV and tuberculosis, access to these medications remains highly constrained in most developing countries [1]. Crowded public health facilities, drug stock outs and lengthy travel times have all been noted as barriers to improving access to effective medications in public sector health facilities [2-5]. It is no wonder, then, that the private sector is typically the first choice in treating common health conditions such as malaria and diarrhea [6]. A major problem, though, is that the latest and most effective medications and technology to conduct effective diagnoses are often unavailable at private drug shops. Many factors likely contribute to low availability of new drugs in retail shops. Retail prices have been shown to impact shop level decisions to stock particular anti-malarial medications in Kenya [7]. Research has indicated that shops sometimes stock specific medications in response to customer demands, rather than policy recommendations [8]. Sellers’ knowledge of pharmaceutical products and proper dosages has been shown to be low in some contexts and could compromise efforts to introduce new products into retail markets [9]. Expanding access to safe and effective medications, along with basic diagnostics for common conditions such as rapid diagnostic tests (RDTs) for malaria, in privately owned drug shops might allow households to receive proper treatment for life threatening diseases earlier, reducing the chance of severe disease and mortality.

**Figure 1:**
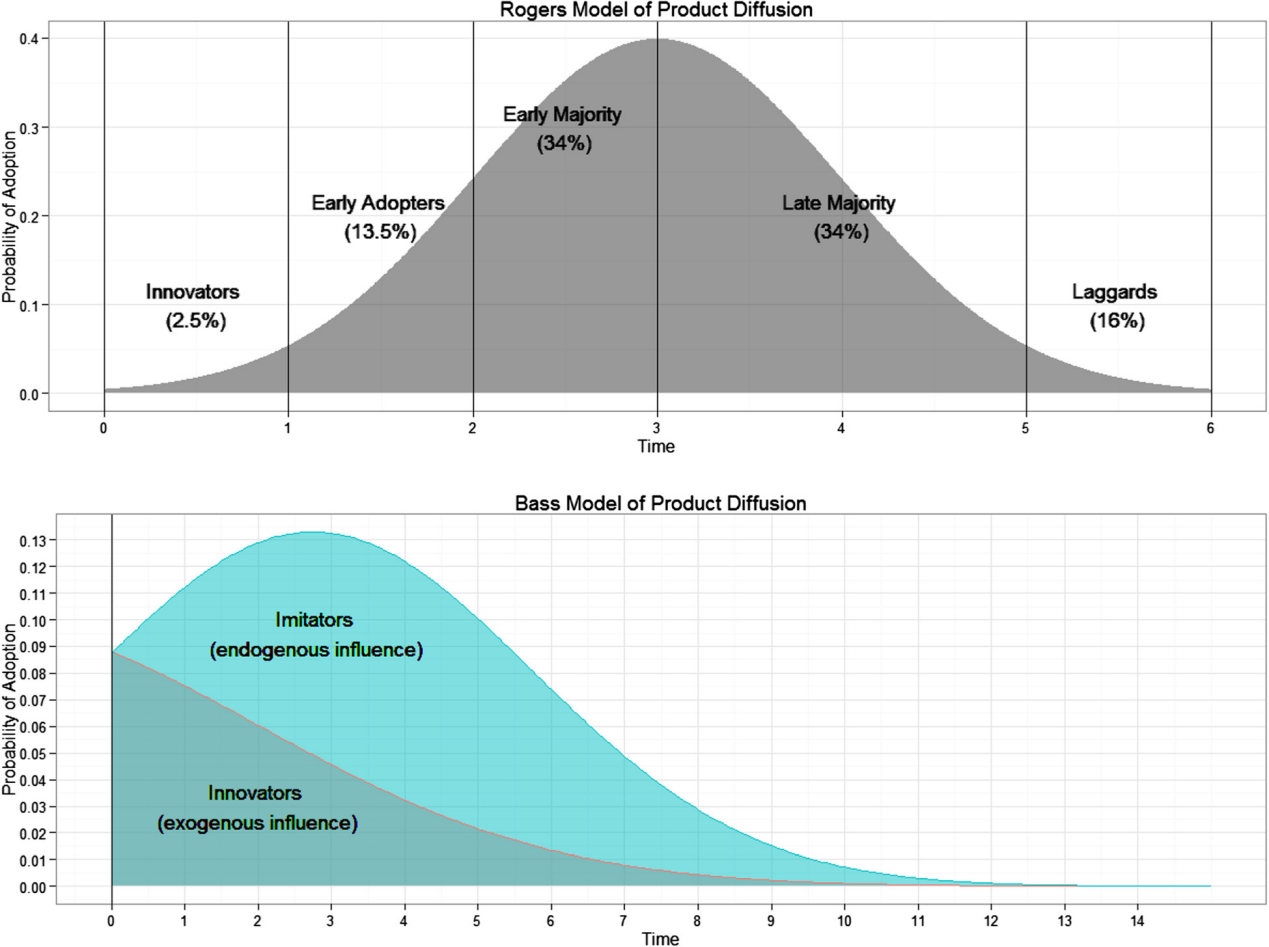
**Models of product diffusion. top)** Rogers’ original model of early to late adopters of new technologies and products **bottom)** The classic Bass model of product diffusion. Innovators spearhead new products at any time, while imitators follow the example of others within the group.

Larger scale development of new technologies for global health in the last decade implies that many new health technologies will be introduced across developing countries in the next few years [10]. Some of these technologies, such as new anti-diarrheal drugs, will sell in the same retail markets as those used for malaria medicines. It therefore becomes important to assess how these technologies will diffuse in the market, which drug shops are most likely to stock them first, and the impact of different variables (such as number of employees, number of customers, sourcing pattern and competition) on the rate of diffusion. Understanding diffusion has important strategic value in order to ensure the rapid and widespread adoption of these health technologies. Diffusion has been studied extensively in developed country markets [11]. In his theory of the diffusion of innovations, Rogers introduces the concept of new “adopters.” Early adopters make use of technologies and subsequently encourage the “late majority” and “laggards” to adopt until the new technology or product spreads through the entire market [12]. Rogers’ model assumed that first adopters of new products differed from people who adopt at later times. Bass, however, expanded up Rogers work by hypothesizing that “innovators” lead the introduction of new products, while “imitators” are inspired to purchase new goods following the example of those around them [13]. He created a mathematical model of adoption and applied it to a range of durable consumer goods. The interpretation of Bass’ “innovators vs. imitators” has been since generalized to address marketing mechanisms of broad advertising vs. social contagion. Since that time, the Bass model has been applied extensively to determine how internal and external factors, such as within group market competition vs. broad media messages, influence product diffusion [14-16]. Bass’ basic ideas work well with current social network theory, where high profile and well-connected individuals influence proximal contacts, who in turn imitate one another [17,18]. See Figure 1 for a graphical representation of both the Rogers and Bass models of product diffusion. Where the Rogers’ model divides adopters on the basis of timing alone, the Bass model assumes that adopters are intrinsically different and may adopt new technologies at any time, but in distinct ways. “Innovators” will choose to adopt new technologies independently of other individuals within the same group, while “imitators” will accept the new products or technologies in response to others around them.

Although much has been done to understand the adoption and diffusion of health technologies in developed countries, little attention has been paid to this in Sub-Saharan African countries. Even less is known for health products which diffuse through retail markets. Market phenomenon are driven by social, economic, and cultural factors which vary significantly between developed countries and low income countries in Sub-Saharan Africa. One study, though, indicated that developing countries are known to adopt new technologies more slowly than developed countries once available, but that diffusion of products within countries occurs quickly [19]. Other studies found, however, that average penetration potential in developing countries is one third as that of developed countries, that peak sales in developing countries take longer than in developed countries, and that delayed product introduction tends to not have positive effects on the overall adoption rate [20]. High prices have been associated with a reduced rate of diffusion of prescription medications in developing countries [16]. Studies of pharmaceutical product diffusion in developed country settings indicated that large health facilities tend to lead the market in adopting new drugs [21]. Some work on supply chains for malaria drugs in developing countries has suggested that the market for drugs in developing country markets is highly complex [22], making studies of diffusion in developing economies difficult. Of secondary interest to the authors of this paper is how products diffuse through networks of shops, connected either through perceived mutual competition, or through common suppliers. A network based approach similar to techniques used in social network analyses was used to analyze competition between shops in the study areas. Network analysis has recently been used to study problems of social networks, such as friend networks for information sharing or large and intricate assemblages of partnerships as applied to the study of sexually transmitted diseases [23-26]. Some work has been done applying network methodologies to economic questions [27-29]. We are unaware of any work that has been done that analyzes network based competition to understand stocking behavior of certain goods and/or pricing.

Knowing how and whether existing models of product diffusion can be applied to drug shops and private markets in sub-Saharan Africa could help public health groups more effectively expand access to essential medications. By identifying specific characteristics of early adopters, new drugs may be introduced more efficiently into the market, thus maximizing availability and minimizing time to universal adoption. We hypothesize that stocking of new products will be the result of a combination of factors which include both local demand for effective products, and a desire for shops to remain competitive in the market by emulating the behavior of peer shops.

In this paper, we leveraged data from a study which longitudinally tracked the adoption of artimisinin combination therapies (ACTs), an effective anti-malarial drug, in Tanzanian Accredited Drug Dispensing Outlets (ADDOs). ADDOs are the lowest level of drug shops that currently have approval from the Tanzania Food and Drug Authority (TFDA) to sell formal anti-malarial medications such as ACTs. The ADDO system was created to improve access to affordable medicines and services in retail pharmaceutical outlets in underserved rural and peri-urban areas [30]. The study was conducted as a part of an operational research project to assess the Affordable Medicines Facility – Malaria (AMFm), an ACT subsidy program hosted by the Global Fund to Fight Tuberculosis, HIV and Malaria. The AMFm provided a supply side subsidy to reduce prices and to increase availability of ACTs in private drug shops in eight pilot countries. Data were collected close to when AMFm subsidized ACTs first became available to ADDOs in Tanzania. Prior to our study, ACTs were prohibitively expensive and generally unavailable in private drug shops [31]. We emphasize that studies of product diffusion usually model the pattern of adoption of new technologies or products into a particular market. At the time of our study, however, ACTs had already been available in the public clinics and hospitals and thus not new to Tanzania. High prices of ACTs, however, heavily constrained availability in private sector outlets, confirmed by the initial round of our survey (see Results). Given our focus on a specific class of private sector drug outlets (ADDOs), the near unavailability of ACTs in these shops and the presence of competing anti-malarial medications, we feel that models of product diffusion as presented in this paper are relevant and applicable. Though the patterns of diffusion and characteristics of early and late adoption might be different for an entirely new product to Tanzania, we feel that this data set offers a unique opportunity to follow a specific product (low cost ACTs) from time of introduction in a specific and important pharmaceutical sector (ADDOs).

This paper will be structured as follows. First, we will describe patterns of ACT stocking over time. Second, we will test associations of competition and demand variables with ACT stocking over the study period. Third, we will characterize shops as early and late adopters and test associations of possible determinants with adopter status. Finally, we will measure and test the contributions of internal and external influence using a mixed influence model. A broad goal of this paper will be to discover whether new product adoption in ADDOs is the result of a shop level motivation to stock ACTs due to actual or yet to be realized customer demand (innovator), or whether shops stock new products due to influence by market competitors.

## Methods

### Ethical approval

The study was approved by the Institutional Review Board of Harvard University School of Public Health (Protocol #19372-102) and the National Institute of Medical Research of Tanzania (NIMR/HQ /R.8a/Vol. IX/1017).

### Data

**Figure 2:**
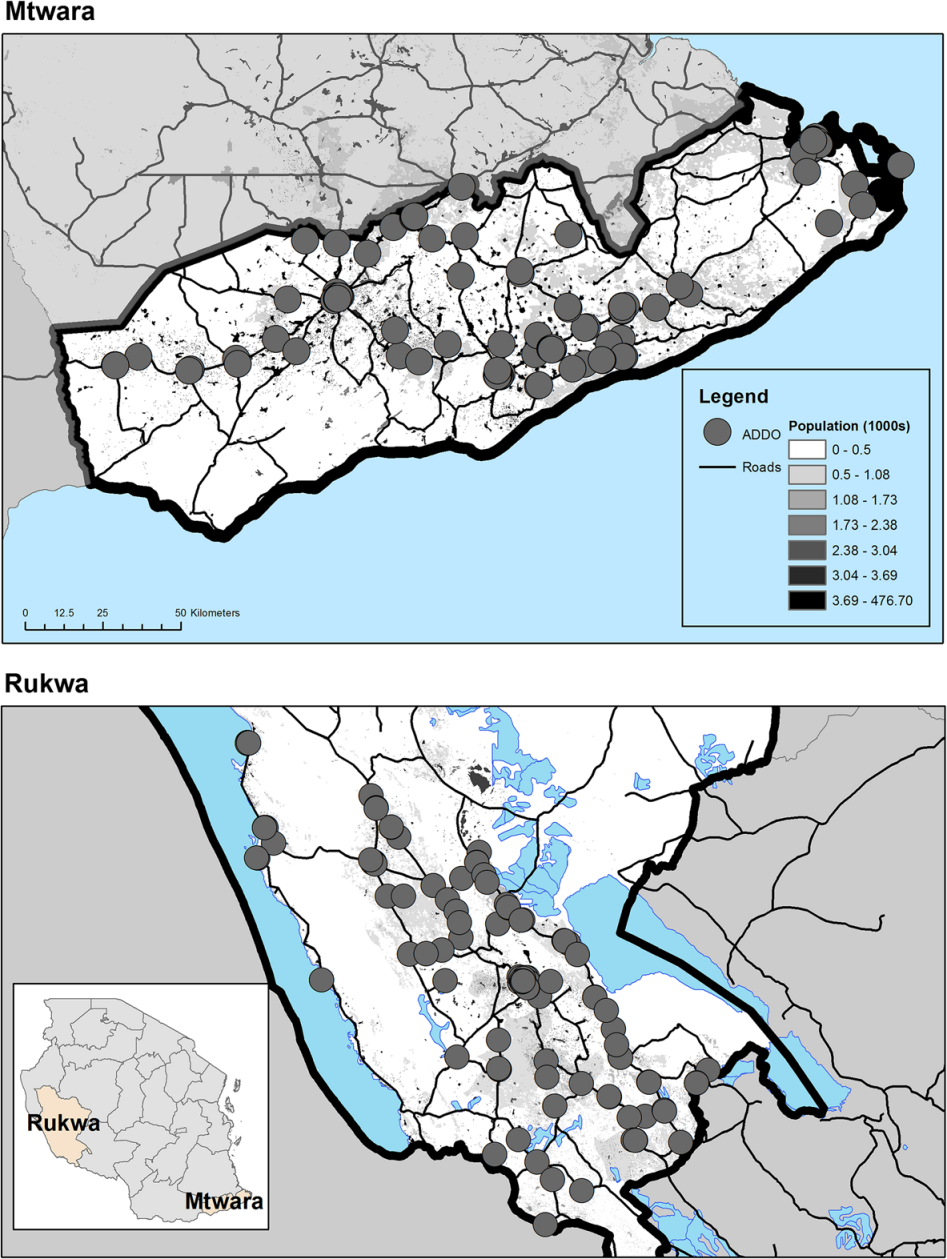
Locations of surveyed ADDOs and target regions.

Two remote, malaria endemic regions of Tanzania (Mtwara and Rukwa) were selected for inclusion in this study in consultation with Tanzania’s Malaria Control Program (NMCP). A complete census of ADDOs was conducted in both regions. Shops were included in the study, contingent upon the availability and verbally confirmed consent of the shop owner and/or dispenser. Over 97% of shops in Mtwara and 99% of shops in Rukwa consented to participate. Community members in one location accused survey teams of practicing witchcraft forcing one shop to drop out of the study. In total, 356 shops were included. (see Figure 2). Data were collected from February 2011 to May 2012 over seven survey periods (see Table 1). Though the first shipment of AMFm ACTs arrived in Tanzania in November 2010 [32], distribution in the private sector did not occur until several weeks later. The beginning of the survey was timed to assess AMFm ACT stocking within a short time of having been first ordered by shops from suppliers. Surveyors noted whether AMFm co-paid ACTs were being stocked on the day of the survey (confirmed by noting the presence of a specialized ACT leaf logo printed on the packaging) and recorded retail prices. For the purposes of this research, “ACT” will refer to ACTs distributed under the AMFm and “ACT stocking” will refer the presence of absence of AMFm labelled ACT products. Comprehensive retail audits, where a full inventory of all anti-malarial products was performed, were administered twice during the study period. At the final comprehensive audit, shop attendants were asked a set of questions regarding sources of supply, stocking frequency, restocking amounts and other questions regarding the business. Though not included in this study, the presence or absence of other essential medications was noted during the comprehensive retail audit.

**Table 1:** Data collection schedule

	**2011**				**2012**		
**Retail Audits**	mid Feb to end Mar 2011	end Mar to mid Apr 2011	end Apr to mid May 2011	Aug 2011	Jan 2012	end Mar-mid Apr, 2012	May 2012
	R1	R2	R3	R4	R5	R6	R7
	**Comprehensive Audit**						**Comprehensive Audit**

### Measurement

Shop level characteristics were assessed using characteristics related to demand, competition, and shop staffing, sourcing and ordering.

#### ***Demand measures***

Demand side characteristics were assessed through a combination of methods. The size of shops’ customer base and possible catchment populations were estimated using data from the survey and available GIS layers. AfriPop has created interpolated grid maps of population for nearly all sub-Saharan African countries using available census data, and satellite imagery [33]. To estimate catchment populations of each ADDO, a radius of 5 km was drawn around the known location of each shop. Total population within 5 km of each shop was extrapolated from the AfriPop population raster for Tanzania and added to the database. When estimating catchments, it was assumed that customers would travel no more than 5 km to obtain goods, but could choose any number of proximal shops. As data on customer preferences was not available, no attempt was made to determine shops of choice. Of interest, rather, was the size of the potential customer base available to each shop. Catchments, particularly in urban areas, could overlap. In addition to population based demand estimates the shop attendants were asked to provide an estimate the number of customers that visited their shop on the previous day, the number of customers that presented to buy drugs for malaria-like conditions, and the number of customers on busy and slow days.

#### ***Competition measures***

Retail competition was analyzed using two methods. First, we utilized a spatial methodology assuming that proximity to other shops implies competition for customers in overlapping catchments [5]. For each shop in the survey, using the exact locations, we calculated the distance to nearest ADDO and the number of ADDOs within 3 km. We performed the same procedure using the locations of known public health facilities offering reproductive and child health (RCH) services^a^.

ADDO representatives were asked to provide the names of up to three other drug shops which they regarded as their competitors. The names were compared with the master list of participating ADDOs in this study and numerical shop keys were assigned for consistency. We constructed a “competition network” of all shops, graphically illustrating the connections between shops. Certain shops may maintain a high level of prominence within the market through having a large market share, the ability to sell products for considerably lower prices than other shops or other factors. Thus, these shops may be more likely to be identified by shops as competitors than other shops, even when distant. Similarly, two shops may be connected to one another through a mutual intermediary, but might be unaware that they are identifying a common competitor. Two basic measures of network centrality were applied in this case: *degree centrality* and *betweenness*. For each shop, the degree is the number of other shops that identify that shop as a market competitor. Betweenness is a measure of whether a specific shop acts as a bridge between otherwise disconnected shops in the network. Calculation of the network measures was performed using UCINET ver. 6 [34].

#### ***Shop staff and stocking habits***

Shops were asked to report the qualifications of dispensers, their level of health training and the number of employees in the shop. Shops were also asked to report the quantity and frequency of replenishment, and the source of obtaining supplies (location of wholesaler).

### Product diffusion under the AMFm

Using Rogers’ (8) methodology, shops were classified into the broad categories of “early adopters” and “late adopters.” This was done through visual inspection of stocking trends. Finer classifications (e.g. “mid to late adopters” and “laggards”) would have been ideal, but it was thought that the small number of survey rounds would not accommodate them.

Coefficients of innovation (internal influence) and imitation (external influence) were produced using a form of the classic Bass diffusion model [13]. The version of the Bass model to describe sales of new products used in this paper is:

$$S\left(t\right)=\frac{e^{-\left(p+q\right)t}}{{\left(1+\frac{q}{p}e^{-\left(p+q\right)t}\right)}^{2}}$$

where S(t) is the rate of change of adoption (at time t) p and q are the coefficients of innovation (the propensity for shops to stock products independent of other shops) and imitation (the propensity for shops to stock products in response to the behavior of peer shops). Parameters were estimated using a non-linear regression methodology [35]. Through these estimates, we hope to quantify the potential roles of shops which indep1endently assume risks of stocking new and untested retail products (innovation) and those which stock as a result of a need to imitate other shops (imitation or social contagion [36]). If p/q ratio, for example, were larger than 1, we might conclude that shops stock ACTs due to endogenous market imitation and a desire to remain competitive, rather than to broad messages sent through either exogenous advertising avenues, messages from common wholesale suppliers or customer demand. The small number of time points prevented the implementation of more rigorous forms of diffusion modeling (e.g. a hazard model with predictive covariates).

### Regression methods/tests of association

Tests for associations of all variables with ACT stocking over the seven survey rounds were performed using a logistic regression model including covariates for survey round and region to account for trend and differences between the two survey areas. We conducted tests for associations of all variables with ACT stocking, and with early/late adopter status. As ACT stocking is likely the result of a combination of factors, some of which may be correlated with one another, a multivariate model of ACT stocking was produced. Backwards selection (based on Akaike’s Information Criterion (AIC)) was used to select an optimal subset set of covariates from all available variables. Variables were eliminated one by one, in order of highest p-value, until a final set of significant covariates which minimized the AIC was reached. Associations of potentially predictive covariates and adopter status were tested using chi-square tests for categorical variables and t-tests for continuous variables. All statistical analyses were performed using R version 2.12.0 [37].

## Results

### Descriptive results

Descriptive results can be found in Table 2.

**Table 2:** Descriptive results of ADDO survey: shop level characteristics

		**Mean/%**
**N**		356
**Competition**		
	**Distance to Nearest ADDO**	3.17 (Range:.10,38.89)
	**Number of ADDOs Within 3km**	8.58 (Range:.0, 35)
	**Distance to Nearest RCH Clinic**	2.23 (Range: .02, 130.4)
	**Number of RCH Clinics Within 5km**	1.85 (Range: 0, 7)
	**Degree**	2.37
	**Betweenness**	7.62
**Demand**		
	**Population Within 5km**	12090
	**Number of Customers on Previous Day**	25.05
	**Number of Customers Presenting for Malaria on Previous Day**	4.56
	**Percent Customers Presenting for Malaria**	19.79%
**Supply**		
	**Frequency of Ordering**		
	**Every Week**	5.58%	
	**Twice a month**	29.88%	
	**Once a month**	43.43%	
	**Every Two Months**	11.16%	
	**Every Three Months**	7.97%	
	**Don’t Know/No Set Schedule**	1.99%	
**Amount Ordered**			
	**1 week**	3.59%	
	**2 weeks**	28.69%	
	**1 month**	45.02%	
	**2 months**	12.35%	
	**3 months**	6.77%	
	**Don’t Know**	3.59%	
**Supplier Location**			
	**Dar Es Salaam**	9.16%	
	**Masasi town**	5.58%	
	**Mbeya**	10.36%	
	**Mtwara town**	25.10%	
	**Sumbawanga**	49.80%	
**Qualifications**			
	**Doctor Present**	12.20%	
	**Pharmacist Present**	1.65%	
	**Nurse or Midwife Present**	65.02%	

Means are presented for ordinal and continuous variables. Percentages are presented for categorical variables.

#### ***Customer demand***

There was an average of approximately 12,000 people living around each ADDO, though catchment populations varied widely (Min:63: Max: 37,241). Shops reported a mean number of 25 customers the previous day, 4.5 of which appeared for malaria. Out of all customers seen the previous day, an average of 20% of them appeared with malaria-like symptoms.

#### ***Market competition***

Distances between shops ranged from .1 km to nearly 40km between each shop, with a mean distance of approximately 3 km. There number of shops located within a 3 km radius around each shop ranged from zero to a maximum of 36 shops. Similarly, ADDOs were located proximally to public RCH clinics, with distances ranging from less than 30 meters to nearly 130 km away.

**Figure 3:**
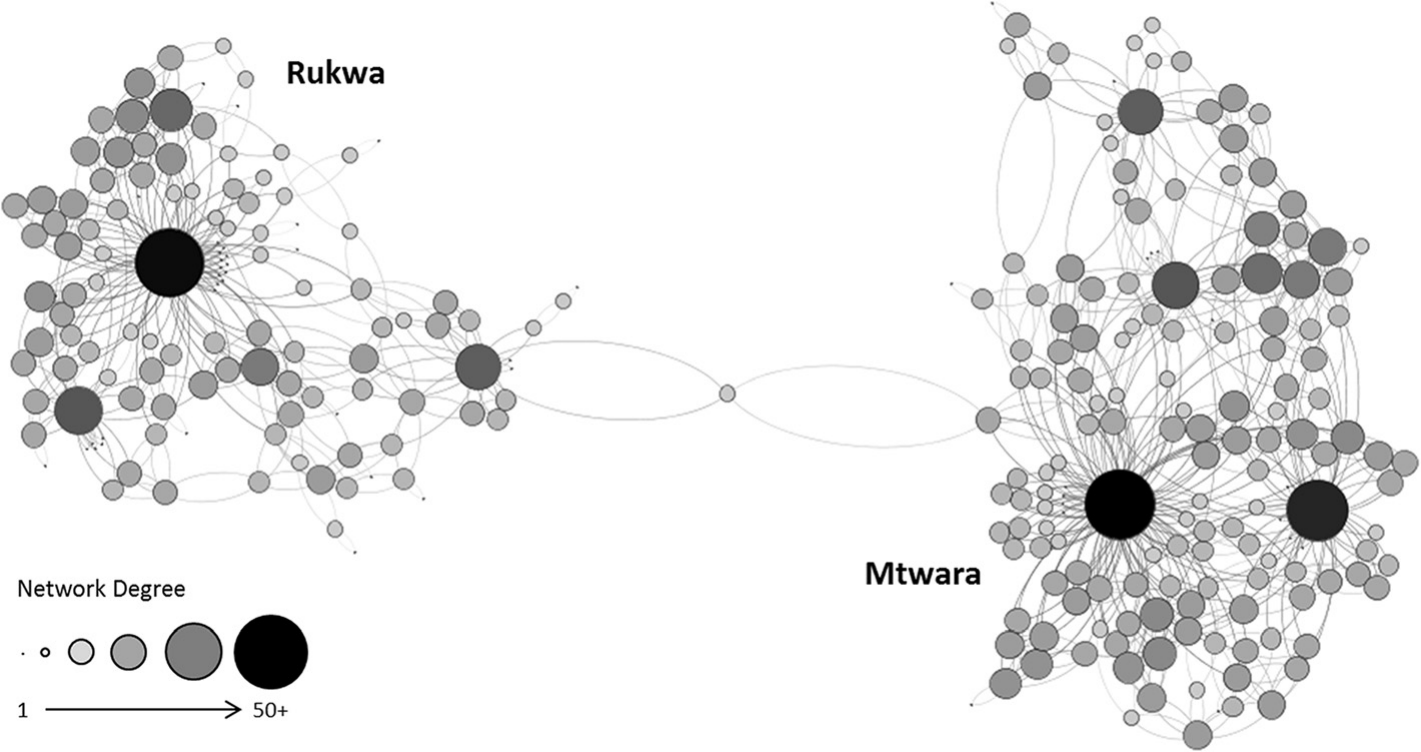
Network of competing shops and common suppliers. Sizes of nodes are proportional to network degree.

Shops were asked to name up to three other shops that were seen as market competitors. Not all shops named a total of three competitors. Many shops reported three competitors as requested, though some reported less. 89% of shops identified at least one competitor. 67% identified at least two, whereas only 44% reported three. From this we were able to construct the network of competing shops and common suppliers (See Figure 3). The mean network degree, or the number of times each shop was named by another, was 2.37 and the average betweenness centrality, a measure of network connectivity, was 7.62. The most highly connected shop had 11 connections and the lowest had 0.

#### ***Shop level ordering practices***

Most shops (43%) reported restocking medicinal supplies once a month. Another 30% order stock biweekly and 12% of shops order stocks once every two months. Few shops ordered stocks weekly and even fewer ordered once every 3 or more months. Upon restocking, most shops reported ordering a month’s supply of drugs or less. Shops generally ordered from proximal wholesalers in nearby mid-sized towns, though nearly 10% reported ordering stocks from distant Dar es Salaam. There was a nurse/midwife present at nearly two thirds of the shops. A few reported having a doctor or a pharmacist on duty.

### Diffusion of ACTs under the AMFm

**Figure 4:**
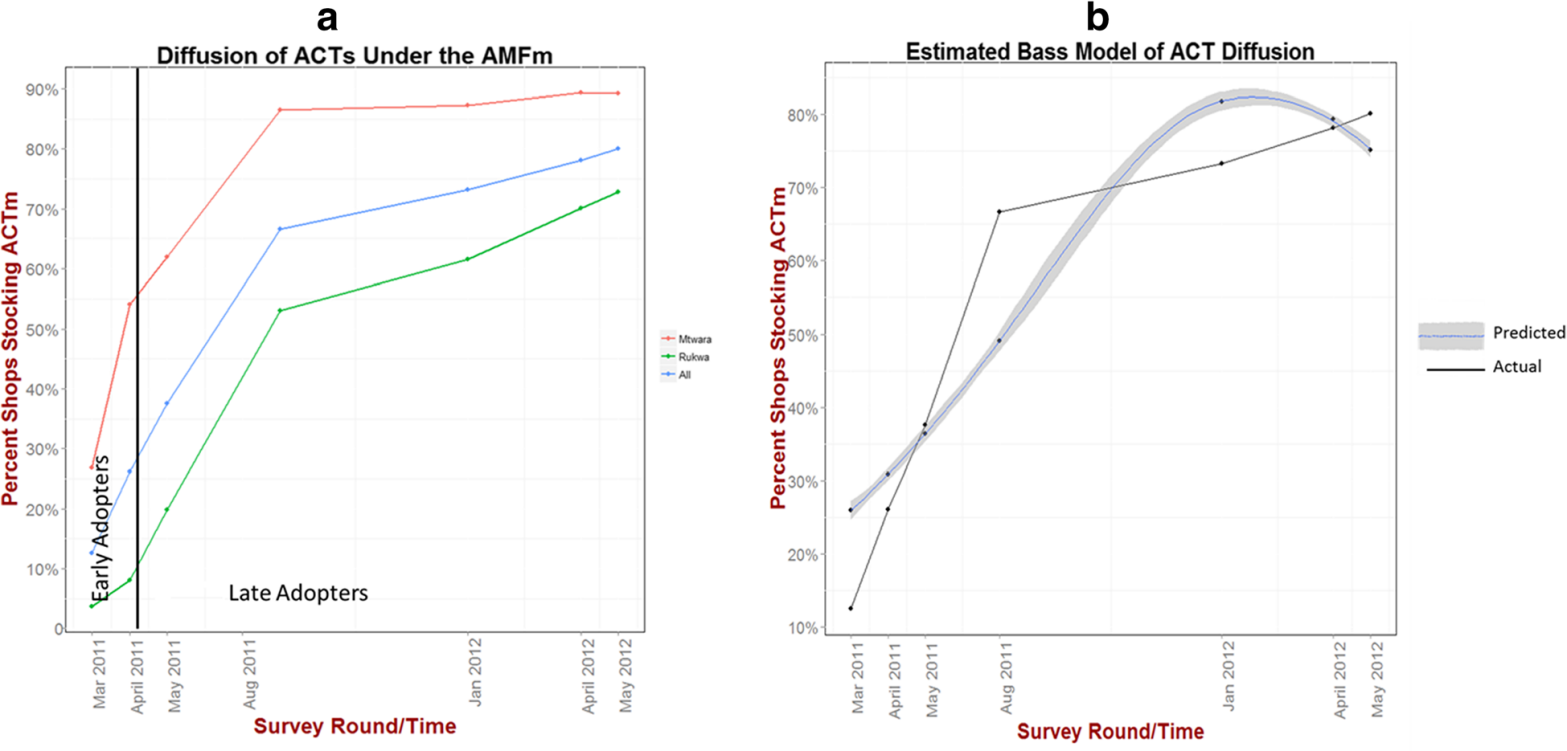
**ACT product diffusion over the seven survey round under the AMFm. a)** Trends by the two survey regions (Rukwa and Mtwara) along with overall trends are plotted. Early adopters are designated as those shops which stocked ACTs in the first two survey periods. All other are classified as mid-to-late adopters. **b)** Bass model of ACT diffusion estimated through non-linear methods.

Stocking of AMFm subsidized ACTs increased rapidly over the seven survey rounds. 12% of shops were reported to have ACTs in stock in the first round. By the final round, more than 80% of shops were stocking ACTs. Patterns of stocking over time were similar between Rukwa and Mtwara, but shops in Mtwara were more likely to stock ACTs overall. (See Figure 4 and Table 3).

**Table 3:** Percent of shops stocking AMFm co-paid ACTs by survey round and region

	**R1**	**R2**	**R3**	**R4**	**R5**	**R6**	**R7**
**Both Regions**	12.55%	26.09%	37.55%	66.67%	73.25%	78.14%	80.08%
**Rukwa**	3.45%	7.64%	19.26%	51.82%	62.41%	70.14%	73.38%
**Mtwara**	24.55%	50.46%	61.76%	87.63%	88.24%	89.32%	88.78%

### Determinants of ACT stocking

Logistic regression models controlling for survey round and region indicated that the number of ADDOs (OR 1.02 (1.01, 1.03)) and RCH clinics within a 3 km radius (OR 1.06 (1.00, 1.12)) were both associated with increased odds of stocking ACTs over the seven survey rounds. Spatial proximity to other ADDOs (OR 1.00 (0.99, 1.00)) and RCH clinics (OR 1.00 (0.98, 1.01)) was not associated with ACT stocking.

While the association of increased population with ACT stocking was not significant (OR 1.01 (0.99,1.02)), increased numbers of customers appearing on the previous day (OR 1.02 (1.00,1.03)) and increased numbers of customers presenting for malaria concerns (OR 1.07 (1.04,1.09)**)** were both predictive of ACT stocking in any round. The fraction of customers presenting for malaria concerns was highly predictive of ACT stocking (OR 3.01 (1.41, 6.46)). Frequency of ordering and supplier location was found not to be associated with ACT stocking. Though the number of shops which had a pharmacist on duty was small, the odds of ACT stocking in shops which employed one were more than three times higher than those which did not when controlling for survey round and region. See Table 4 for complete results.

**Table 4:** Odds ratios and confidence intervals for bivariate associations of shop level characteristics with AMFm co-paid ACT stocking controlling for trend over the seven survey rounds and survey region

		**OR (95% CI)**	**p**
**Competition**			
	**Distance to Nearest ADDO**	1.00 (0.99,1.00)	0.23
	**Number of ADDOs Within 3km**	1.02 (1.01,1.03)	0.001
	**Distance to Nearest RCH Clinic**	1.00 (0.98,1.01)	0.90
	**Number of RCH Clinics Within 3km**	1.06 (1.00,1.12)	0.04
	**ACT Stocking by one or more named competitors**	1.51 (1.17, 1.95)	.002
	**Degree**	1.16 (1.08,1.25)	<.0001
	**Betweenness**	1.01 (1.00,1.01)	0.01
**Demand**			
	**Population Within 5km**	1.01 (0.99,1.02)	0.31
	**Number of Customers on Previous Day**	1.02 (1.00,1.03)	<.0001
	**Number of Customers Presenting for Malaria on Previous Day**	1.07 (1.04,1.09)	<.0001
	**Percent Customers Presenting for Malaria**	3.01 (1.41,6.46)	0.004
**Supply**			
**Frequency of Ordering**			
	**Every Week**		
	**Once a month**	1.01 (0.59,1.73)	0.96
	**Twice a month**	1.13 (0.65,1.96)	0.66
	**Every Two Months**	0.89 (0.48,1.65)	0.70
	**Every Three Months**	0.65 (0.33,1.28)	0.21
	**Don’t Know/No Set Schedule**	1.85 (0.72,4.77)	0.20
**Amount Ordered**			
	**1 week**		
	**2 weeks**	0.71 (0.37,1.38)	0.31
	**1 month**	0.69 (0.36,1.32)	0.26
	**2 months**	0.70 (0.35,1.43)	0.33
	**3 months**	0.33 (0.15,0.74)	0.01
	**Don’t Know**	0.59 (0.25,1.40)	0.23
**Supplier Location**			
	**Dar Es Salaam**		
	**Masasi town**	0.88 (0.45,1.74)	0.71
	**Mbeya**	1.93 (0.92,4.06)	0.08
	**Mtwara town**	1.54 (0.93,2.53)	0.09
	**Sumbawanga**	1.22 (0.64,2.33)	0.55
**Qualifications**			
	**Doctor Present**	1.06 (0.76,1.48)	0.74
	**Pharmacist Present**	3.12 (1.29,7.53)	0.01
	**Nurse or Midwife Present**	1.20 (0.91,1.60)	0.20

Shops were asked to list up to three of their perceived competitors. A logistic regression model for ACT stocking was produced testing associations with the stocking status of perceived competitors while controlling for survey round and region. Tests indicated that the odds of stocking ACTs for shops where one or more named competitors also stocked them were 1.51 (CI: 1.17, 1.95) times higher than shops whose competitors did not stock ACTs. Increasing network degree of shops was highly associated with increased odds of ACT stocking (OR 1.16 (1.08, 1.25)). Betweenness centrality was statistically associated with ACT stocking (OR 1.01 (1.00, 1.01)), though the effect was weak.

**Figure 5:**
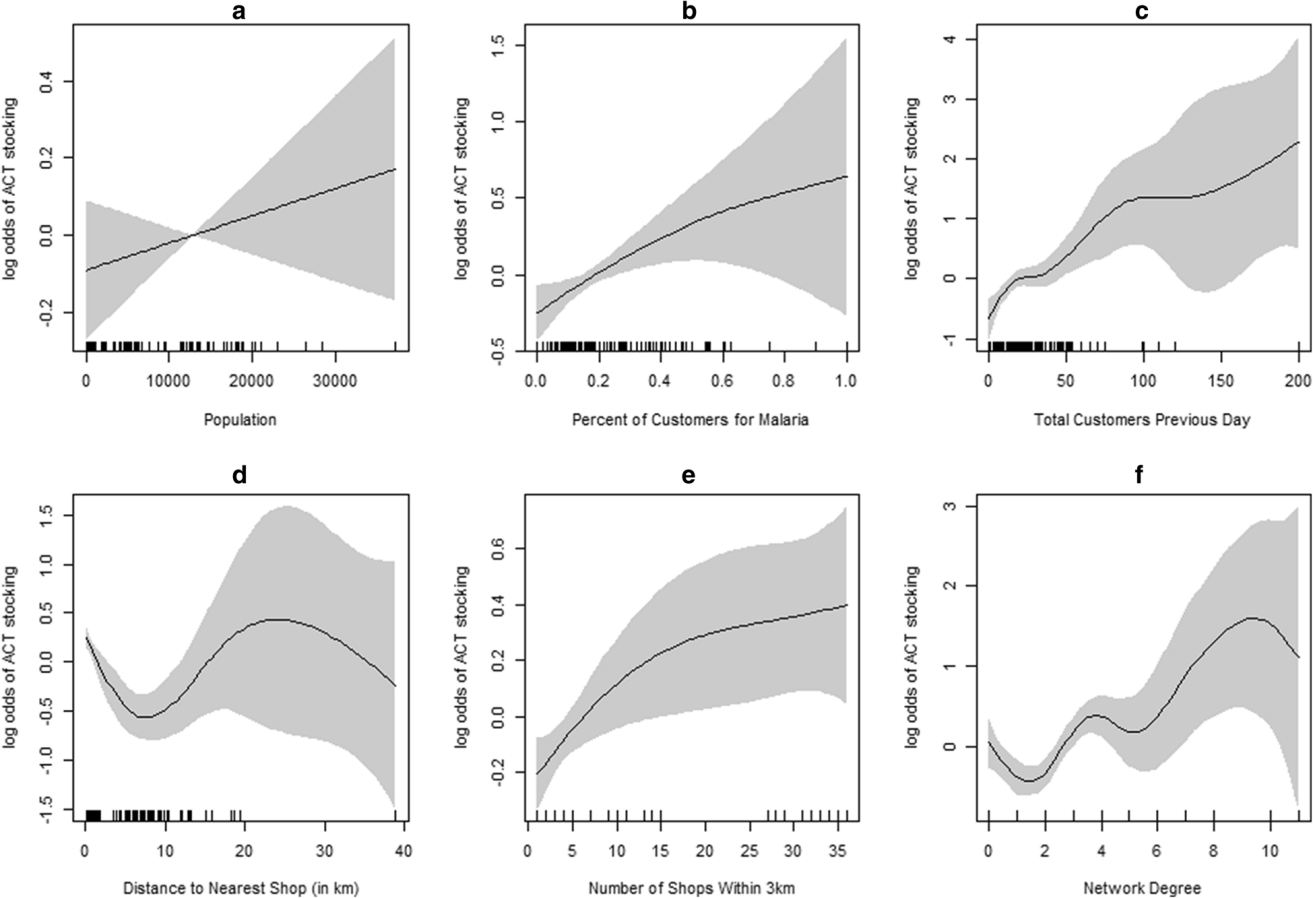
**Plots of smoothed estimates of predictive covariates on the log odds of stocking AMFm ACTs controlling for survey round and region. a)** population, **b)** percent of customers presenting for malaria concerns, **c)** total customers the previous day, **d)** distance to nearest shop, **e)** number of shops within a 3km radius and **f)** network degree.

As the relationship between predictors and outcomes may not be entirely linear, patterns of association of predictors on ACT stocking were explored graphically. Smoothed estimates of predictive covariates controlling for survey round and region can be seen in Figure 5. Population, percent of customers presenting for malaria concerns, number of shops within 3km, and total customers on the day of survey were all positively and mostly linearly associated with ACT stocking. Distance to nearest shop was inversely associated with ACT stocking for shops which were very proximal to one another (under 10 km), but not associated at all for very isolated shops. Network degree showed positive associations with ACT stocking.

#### ***Multivariate model of ACT stocking***

All variables from Table 4 and the variable of ACT stocking by one or more named competitors were considered for the model. The final model, produced through a backward selection procedure using AIC, is presented in Table 5. Controlling for region and survey round, the number of customers on the previous day and the fraction of customers appearing for malaria concerns were both positively associated with ACT stocking. Increased numbers of proximal shops was associated with a statistically significant increase in the likelihood of stocking ACTs. Larger numbers of proximal RCH clinics was associated with a decreased likelihood of stocking ACTs. In short, ACT stocking appeared to be associated with a combination of shop traffic and the presence of proximal competitors.

**Table 5:** Results of optimal multivariate model based on a backward selection procedure

	**OR (95% CI)**	**p**
**(Intercept)**	0.14 (0.09, 0.24)	<.0001
**Total number of customers on previous day**	1.02 (1.01, 1.02)	<.0002
**Percentage of customers appearing for malaria concerns (per 10% increase)**	1.19 (0.52, 2.70)	<.0003
**Number of ADDOs within 3km radius**	1.04 (1.02, 1.07)	0.001
**Number of RCH clinics within 3 km**	0.89 (0.78, 1.01)	0.065
**Round 1**	Ref	
**Round 2**	2.72 (1.61, 4.61)	<.0001
**Round 3**	4.99 (2.96, 8.43)	<.0001
**Round 4**	22.55 (13.07, 38.90)	<.0001
**Round 5**	28.41 (16.40, 49.21)	<.0001
**Round 5**	41.73 (23.78, 73.24)	<.0001
**Round 7**	49.06 (27.58, 87.26)	<.0001
**Mtwara**	Ref	
**Rukwa**	0.15 (0.11, 0.20)	<.0001

All variables presented previously were included in the model, and then removed one by one in order of highest p-value until an optimal set of significant variables (with minimum AIC) was reached.

### Early/late adopters

Shops which first stocked ACTs in any of the first two survey rounds were considered “early adopters” while all other shops were classified as “late adopters.” Though there was evidence that some measures were associated with the probability of stocking in any round, very few of our measures were associated with early vs. late adopter status. Early adopters, however, tended to be located in areas of higher population than that of late adopters. The fraction of customers presenting for malaria concerns was also slightly higher in early adopting shops compared to shops which started stocking ACTs at a later time (24% vs. 18%). The location of the wholesale supplier was significantly associated early adoption of ACTs. More than half of shops which first stocked ACTs within the first three rounds reported buying their supplies from a wholesale supplier in Mtwara perhaps indicating that this particular supplier offered subsidized ACTs ahead of other wholesalers. Full results can be seen in Table 6.

**Table 6:** Associations of shop level characteristics with early and late adopters of AMFm ACTs with tests for statistical differences

		**Early Adopter**	**Mid/Late Adopters**	**p**
**Competition**				
	**Distance to Nearest ADDO**	2.65	5.43	0.23
	**Number of ADDOs Within 3km**	7.76	9.41	0.22
	**Distance to Nearest RCH Clinic**	1.55	2.79	0.18
	**Number of RCH Clinics Within 3km**	1.57	1.95	0.16
	**Degree**	2.38	2.42	0.86
	**Betweenness**	9.36	7.21	0.57
**Demand**				
	**Population Within 5km**	15238.84	11984.37	0.02
	**Number of Customers on Previous Day**	28.36	24.76	0.41
	**Number of Customers Presenting for Malaria on Previous Day**	5.38	4.38	0.19
	**Percent Customers Presenting for Malaria**	0.24	0.18	0.01
**Supply**				
**Frequency of Ordering**				0.68
	**Every Week**	4.05%	6.85%	
	**Once a month**	47.30%	38.36%	
	**Twice a month**	29.73%	30.14%	
	**Every Two Months**	8.11%	13.01%	
	**Every Three Months**	9.46%	8.90%	
	**Don’t Know/No Set Schedule**	1.35%	2.74%	
**Amount Ordered**				0.3
	**1 week**	1.35%	4.79%	
	**2 weeks**	28.38%	26.71%	
	**1 month**	52.70%	41.10%	
	**2 months**	9.46%	13.70%	
	**3 months**	6.76%	8.22%	
	**Don’t Know**	1.35%	5.48%	
**Supplier Location**				<.0001
	**Dar Es Salaam**	10.81%	8.90%	
	**Masasi town**	9.46%	4.79%	
	**Mbeya**	4.05%	13.70%	
	**Mtwara town**	54.05%	12.33%	
	**Sumbawanga**	21.62%	60.27%	
**Qualifications**				
	**Doctor Present**	9.09%	17.30%	0.14
	**Pharmacist Present**	1.29%	2.56%	0.88
	**Nurse Present**	77.92%	72.43%	0.45

**Figure 6:**
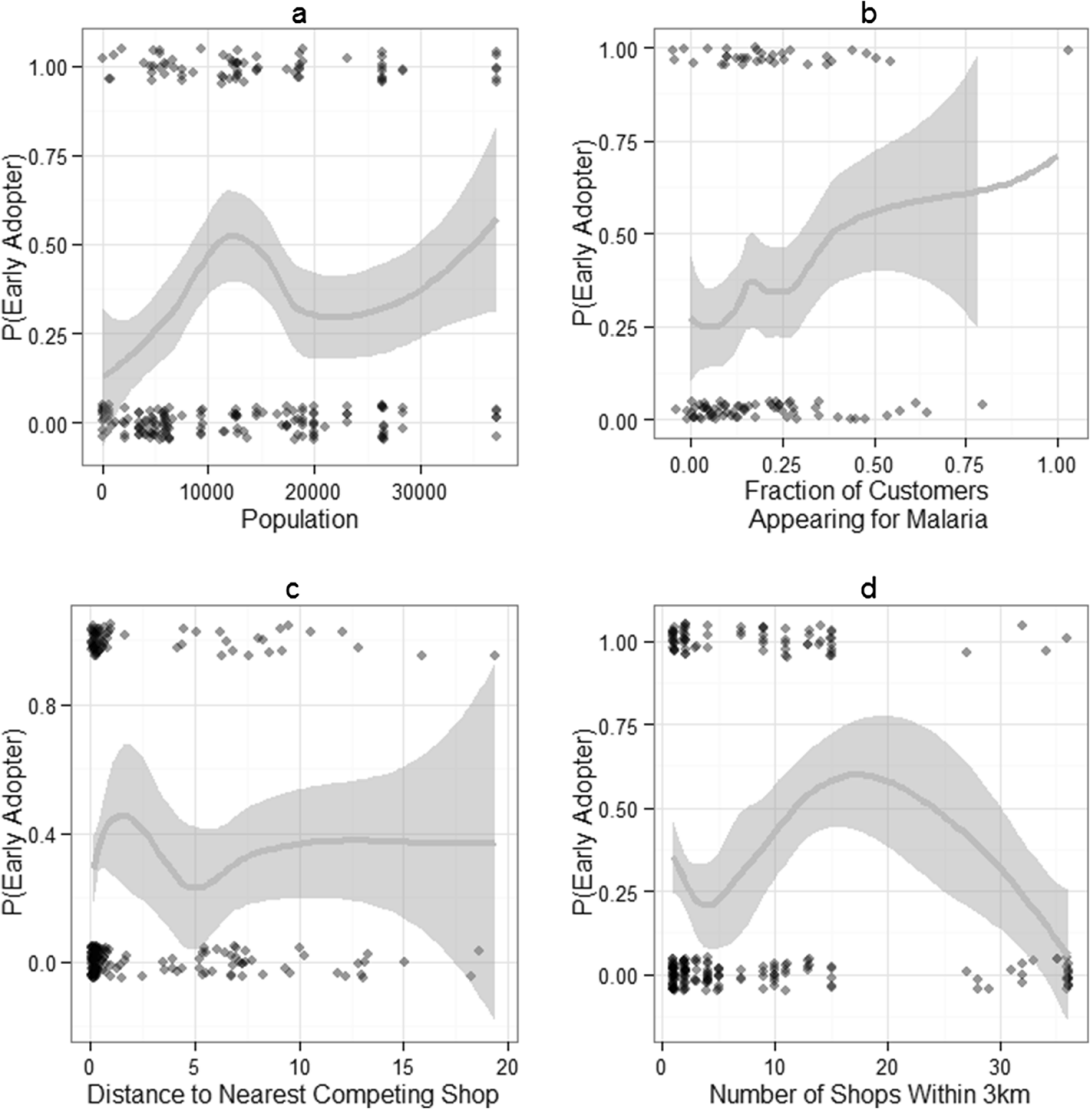
**Probability of being an early adopter of ACTs under the AMFm by a) population, b) network degree, c) distance to nearest competing shops and d) the number of shops within a 3km radius.** Trend lines are produced using loess fitting. Points represent adopter status of shops.

Graphically, we sought to characterize adopter status by population, distance to nearest competing shop, and the number of shops within a 3km radius (see Figure 6). Increasing population and increased fraction of customers appearing for malaria concerns was associated with an increased probability of being an early adopter. Distance to nearest competing shops was only relevant for shops where the closest shop was within 5km. Past 5km, there appeared to be no association with adopter status. Increased numbers of proximal shops (within a 3km radius) was associated with an increased probability of being an early adopter. However, shops located within very dense and competitive areas appeared to have the lowest probability of stocking ACTs under this program.

### Internal vs. external influence

Again, the small number of time points constrained our ability to estimate coefficients of internal and external influence through more complex models. However, using the previously presented model form, parameters estimates for the Bass model were p=.01, q=.53 yielding a p/q ratio of 53. Shops may be responding to broader market trends (imitative behavior) when making decisions to stock ACTs, as opposed to a desire to satisfy local demand.

## Discussion

An operational research study of the AMFm provided us with an opportunity to track the diffusion of a health product in the private market in a developing country from its introduction to widespread availability. We know of no other study which has focused on the question of how pharmaceutical products diffuse in developing world markets using a comprehensive longitudinal series of surveys. Understanding the specific characteristics of shops which stock new products and shops which adopt new products earlier than others could help inform targeting and design of future campaigns to increase availability.

Through our study, we found that busier, more prominent shops in direct competition with others in densely populated areas were more likely to stock ACTs than isolated shops which served fewer customers. Another study also found that shops proximal to outlets which also sold ACTs were more likely to stock them, confirming that shop level competition is a factor in the stocking of new medicinal products [38]. We also found evidence that might suggest that shops which have a higher fraction of their customers presenting for malaria concerns are more likely to stock anti-malarial medications overall and are more likely to adopt new products targeting malaria more quickly. We noted that a specific wholesaler location (Mtwara) was associated with early product availability in small shops suggesting that wholesalers also may play an important role in expanding product availability. Though the limited number of time points prevented more rigorous analysis using a mixed influence model, the parameters estimates from the Bass model used suggest that shops predominantly imitate one another when stocking ACTs, rather than respond exclusively to customer demands. This result is to be expected if shops are responding to suggestions from wholesalers to stock new products. An optimal multivariate model included a combination of increased customer demand, both overall and for malaria, and increased numbers of proximal sources of pharmaceutical goods. This could imply that decisions to stock new products are the result of a complex mix of factors related to both a need to imitate and thus remain competitive in the market and to simultaneously satisfy the potential demands of customers. More work needs to be done to parse out the relative impacts of competition and demand factors in influencing shop level decisions to stock new products.

Our study suffered from many limitations. First, the ability to generalize our results to other regions of Tanzania and to other Sub Saharan African countries may be limited. Rukwa and Mtwara respectively represent inland and coastal areas of Tanzania in terms of access to public and private health care. The results might differ in the area surrounding heavily urbanized Dar es Salaam. Follow up research might include urbanized Dar es Salaam to determine whether patterns of diffusion differ from those in other, more remote regions. Further, presence of the shop accreditation system in Tanzania might limit the ability to generalize our findings to other African countries. Though private, the ADDOs are licensed and accountable to the central regulatory body which, in cooperation with local health authorities and development partners, attempts to ensure standards of shop keeper knowledge and product quality. The private pharmaceutical sector in other Sub Saharan African countries might not be as formalized. Future research might attempt to monitor patterns of diffusion of new pharmaceutical products in private sector health markets in a number of countries simultaneously.

Second, though we had access to detailed data such as shop locations, names of competing shops and customer numbers, we lacked more detailed knowledge of the attitudes and experiences of the shop proprietors themselves. Informal interviews while study teams were in Tanzania indicated that business knowledge and attitudes were heterogeneous among shops. Future studies might seek to collect more detailed data on how shop owners strategize their businesses, assuming they do at all. Knowing more information about what criteria shop owners use to determine which new products will they start stocking might help create efficient strategies for the launch and faster adoption of new health technologies in private markets. There is evidence that educational strategies that seek to improve the knowledge and thus efficiency of small drug providers has been shown to be effective in some contexts [39].

Third, the unique nature of the AMFm might have produced a case of product diffusion that may not always be applicable to other contexts. New products that lack such public support and deep price subsidies might not diffuse as quickly. The AMFm was widely publicized and ACTs were likely known to customers and shopkeepers. Shops may be reluctant to stock a product that is unfamiliar to them or has yet to be accepted by or is unknown to current customers. Also, deeper information on the demographics and experiences of the catchment populations, such as socio-economic profiles or the incidence of fever in the catchment, might have better informed the demand variables. Further country level research might also examine the role of spatial heterogeneities of malaria risk in influencing shop level stocking behavior.

## Conclusions

Decisions to stock new pharmaceutical products in Tanzanian ADDOs are influenced by a combination of factors related to both market competition and customer demand, but are particularly influenced by the behavior of competing shops. Efforts to expand access to new pharmaceutical products in developing country markets could benefit from initial targeting of high profile shops in competitive markets and wholesale suppliers to encourage faster product diffusion across all drug retailers. Our results might be specific to AMFm ACTs, given their subsidized nature and the previous availability of ACTs in the public sector. Thus, future research might follow an entirely new product in a market like Tanzania. Studies of product diffusion in developing country pharmaceutical markets will help inform policy and market based strategies to maximize uptake while minimizing time to adoption, improving access to life saving medications.

## Endnotes

^a^RCH clinics were used as a proxy in place of all public health facilities as we did not have GPS coordinates of all public health facilities. A large fraction of public health facilities offer RCH services so this was a reasonable approximation.

## Abbreviations

ACT: artemisinin combination therapy; ADDO: accredited drug dispensing outlet; AMFm: Affordable Medicines Facility – Malaria; GIS: geographic information systems; RCH: reproductive and child health.

## Competing interests

OS is a member of, and JC receives research support from, the Clinton Health Access Initiative, which is actively supporting efforts to expand funding for and implementation of ACT treatment in the private sector.

## Authors’ contributions

PY, JC, OS, JM, and JA were involved in the conception and design of the overall longitudinal study. PL, PY, JC, SA, and JA were involved in the design and analysis pertaining to research questions addressed in this specific paper. PL performed statistical analyses and was involved in drafting the manuscript SA participated in the design, coordination of the study, analysis, and drafting of the manuscript. OS and JM provided inputs to study design, study context, and drafting of the manuscript. All authors read and approved of the final manuscript.

## Pre-publication history

The pre-publication history for this paper can be accessed here:

http://www.biomedcentral.com/1472-6963/13/526/prepub
